# Spontaneous intra-peritoneal bleeding secondary to warfarin, presenting as an acute appendicitis: a case report and review of literature

**DOI:** 10.1186/1471-2326-6-7

**Published:** 2006-10-11

**Authors:** Jayesh Sagar, Vikas Kumar, Dharmendra K Shah, Ashok Bhatnagar

**Affiliations:** 1 Department of Surgery, Royal Free Hospital, London, UK; 2 Department of Orthopaedics, Princess Alexandra Hospital, Harlow, UK; 3 Department of Surgery, S.S.G. Hospital, Baroda, India; 4 Department of Surgery, Government Medical College, Surat, India

## Abstract

**Background:**

Warfarin is a coumarin anti-coagulant, used widely for the therapeutic and prophylactic anticoagulation. Although, it is considered as a life saving medicine, it is associated with the significant adverse effects including intra-abdominal bleeding, which have been very well documented in literature. However, the presentation of warfarin induced intra-peritoneal bleeding as an acute appendicitis has not been reported in English literature. We report this rare, spontaneous intra-peritoneal bleeding secondary to warfarin therapy, mimicking the signs and symptoms of an acute appendicitis for the first time in English literature.

**Case presentation:**

A 41 year-old female patient who was on warfarin for prophylaxis following the previous episode of pulmonary embolism, presented to the Casualty with the typical symptoms of an acute appendicitis. During operative intervention, we found it to be the spontaneous intra-peritoneal bleeding secondary to warfarin. The patient recovered well following the operation.

**Conclusion:**

We recommend the use of the radiological investigations in all the cases of acute abdomen who are on warfarin even if the INR is within the therapeutic range.

## Background

Warfarin is a coumarin anti-coagulant, widely used for the therapeutic and prophylactic anti-coagulation. Although it is considered as a life saving medicine, it is associated with the several significant adverse effects. Intra-peritoneal bleeding is one of the complications, usually following trivial trauma. There are only very few reported cases of the spontaneous haemoperitoneum in English literature without any evident cause [1,2]. Spontaneous onset of the intra-peritoneal bleeding due to warfarin therapy is also exceptional. We report a case of the spontaneous intra-peritoneal bleeding secondary to warfarin therapy, mimicking the signs and symptoms of an acute appendicitis in a 41-year old Caucasian female, for the first time in English literature. We strongly recommend the consideration of this rare complication in the differential diagnosis of all the cases of acute abdomen in patients who are on warfarin therapy regardless of INR levels, especially in presence of anaemia and tachycardias. We also emphasize the use of the radiological investigations in such cases to achieve the diagnosis to avoid unnecessary surgical intervention.

## Case presentation

A 41 year-old Caucasian female patient was referred to us by general practioner (G.P.) with 2-day history of the migratory abdominal pain. Initially, the pain was constant and dull in the peri-umbilical region, which later became sharp and localised in the right iliac fosssa. She also complained of four episodes of vomiting and loss of appetite. She occasionally felt pain in her right shoulder. There was no history of even trivial trauma. In the past, she had 3 episodes of pulmonary embolism following hysterectomy and she had been on warfarin for the prophylaxis since then. She used to take tylex (paracetamol and codeine), pantoprazole, tramadol, senna, migraleve, zimovane and fentanyl citrate lozenges as her regular medications. She had 35-pack year history of smoking. On examination, she was afebrile (however, temperature of 37.8°C noticed by General Practitioner 6 hours before presentation in the Casualty) but tachycardic with pulse rate of 134/minute. There was no bruising or haematoma in the abdominal wall. The abdomen was distended with marked tenderness in the right lower quadrant. There were guarding and rebound tenderness in the right iliac fossa. The rectal examination revealed tenderness in the right pelvic wall. The urine dipstick was positive for the ketones and trace of protein. The pregnancy test was negative. The blood investigations showed haemoglobin of 10.8 gm/dl with reduced haematocrit of 32%, White Blood Cells of 11.8 × 10^9^/l and INR of 2.2. The blood biochemistry did not reveal any abnormality.

The clinical diagnosis of acute appendicitis was made. She was scoring 8 according to modified Alvarado scoring system [3]. The decision for appendectomy was made and the consent was given for the emergency appendectomy. Through the Lanz incision, about 200 ml of haemorrhagic fluid was aspirated from the peritoneal cavity. The appendix and the mesoappendix were looking normal. The appendectomy was performed. Intra-operatively, there were several blood clots present, so the incision was converted to the formal transverse infra-umbilical laparotomy incision. The large, 10 × 15 cm^2 ^size blood clot was evacuated from the pelvis. Despite thorough search for the active bleeding site, we could not find any cause for active bleeding. The final diagnosis of the spontaneous intra-peritoneal bleeding secondary to warfarin was made. The abdomen was closed with the low suction drain, which drained about 200 ml of the serosanguinous fluid over next 2 days. 2 units of fresh frozen plasma were transfused in the immediate post-operative period. The warfarin was stopped after the operation. The patient was re-warfarinised on 8^th ^post-operative day and was followed up in the surgical and the haematology clinics. At 2 weeks follow up visit in surgical clinic; patient was doing well without any complaints and complications. The histology of the appendix confirmed normal appendix without any inflammation.

## Conclusion

Warfarin is a life saving drug, extensively used in the treatment and the prophylaxis for the various clinical conditions including deep vein thrombosis, pulmonary embolism, valvular heart disease, atrial fibrillation, recurrent systemic emboli, recurrent myocardial infarction, prosthetic heart valves and prosthetic implants [4-6]. However, it is associated with the serious adverse effects such as the haematuria, soft tissue bleeding and haematoma, intra cerebral bleed, skin necrosis, purple toe syndrome and abdominal bleed. Theoretically, the bleeding can occur in any part of the body following any kind of the anticoagulation therapy. Bleeding in the gastrointestinal tract is by far the most common complication of the warfarin therapy. Bleeding may occur intra-, extra- or retroperitoneally [7,8], but the intramural bowel haematoma is the most common cause of the abdominal pain in the patients who are on anticoagulantion therapy [8-10]. It is crucial to differentiate between the intra-peritoneal bleeding and the intra-mural haematoma, as most of the intra-mural bowel haematomas respond to non-operative treatment [11,12]. Here, we report the rare complication of the warfarin therapy – spontaneous intraperitoneal bleeding, mimicking the clinical features of an acute appendicitis. According to our knowledge, such clinical presentation has not been reported previously in English literature.

The two most important determinants of the warfarin induced bleeding is the intensity of therapy and the maximal time in therapeutic range [4]. Bleeding is a major complication in the early phase of the warfarin therapy according to the most studies [4,13]. Bleeding is more likely to occur in the patients with the more intense therapeutic range (INR between 2.5 and 3.5) than in the less intense therapeutic range of warfarin (INR between 2 and 3) [13,14]. However the interesting point in our case is the presentation of this severe intra-peritoneal bleeding in the less intense therapeutic range of warfarin (INR 2.2).

This also raises a question about the present management of acute appendicitis in UK, as we have not yet accepted the CT scan as a mandatory investigation for the diagnosis of appendicitis. Although the modified Alvarado scoring system has been a useful means for the management of an acute appendicitis, we think, the CT scan and/or ultrasound in this case might have confirmed the diagnosis and patient would have been avoided the surgery and been observed with the reversal of anticoagulation [15]. Use of the CT scan may not be possible due to the medical reasons or the fear of over utilization, we recommend the use of the radiological imaging in such cases. The other learning point in this case is the history of occasional pain in right shoulder at time of presentation. This may be due to the blood under the diaphragm causing irritation of the phrenic nerve, causing referred pain in the shoulder (well known as Kehr's sign) but this became evident retrospectively only.

This case provides a learning lesson to the young junior surgeons as well as to other specialists such as general practioners and physicians to consider this rare but significant complication of warfarin in the differential diagnosis of all the cases of acute abdomen/abdominal pain in patients who are on warfarin therapy, even if the INR is in the low therapeutic range. We also emphasize that one must consider intra-peritoneal bleeding in presence of anaemia and tachycardias in patients on warfarin therapy. We recommend the use of the radiological investigations such as CT scan or ultrasound in these cases to achieve diagnosis and to avoid unnecessary surgery.

## Competing interests

The author(s) declare that they have no competing interests.

## Authors' contributions

J S – As a main author, I have made substantial contribution in design and interpretation of this case report. I have drafted the manuscript and amended the manuscript in accordance to the reviewer's comments with intellectual content. I have given the final approval of the version to be published. As a main author, I have made sure that all the contributors have participated significantly in the preparation of the manuscript and given the final approval for publication.

V K – As a co-author, I have helped in reference search and contributed significantly in the background part of the manuscript and given the final approval of the version to be published. I have also contributed in drafting and revising the manuscript in accordance to the reviewer's comments.

D K S – As a co-author, I have helped in reference search and contributed significantly in the case presentation part of the manuscript and given the final approval of the version to be published. I have also contributed in drafting and revising the manuscript in accordance to the reviewer's comments.

A B – As a co-author, I have helped in reference search and contributed significantly in the conclusion part of the manuscript and given the final approval of the version to be published. I have also contributed in drafting and revising the manuscript in accordance to the reviewer's comments.

## Pre-publication history

The pre-publication history for this paper can be accessed here:


